# Association of serum C1q/TNF-related protein-3 (CTRP-3) in patients with coronary artery disease

**DOI:** 10.1186/s12872-017-0646-7

**Published:** 2017-07-28

**Authors:** Shuhong Wang, Yuan Ling, Wei Liang, Linhui Shen

**Affiliations:** Department of Geriatrics, Ruijin Hospital, Shanghai Jiao-Tong University School of Medicine, Shanghai, 200025 China

**Keywords:** Complement C1q tumor necrosis factor related protein 3, Coronary artery disease, Coronary lesioned vessels

## Abstract

**Background:**

Recent studies have demonstrated that complement C1q tumor necrosis factor related proteins (CTRPs) have diverse biological influences on the cardiovascular system. CTRP 3 is a member of the CTRP superfamily, which may play a pivotal role in the pathogenesis of coronary artery disease (CAD). Here, we investigated whether serum levels of CTRP 3 are associated with the prevalence and the severity of CAD.

**Methods:**

In this study, 145 eligible participants were included who underwent coronary angiography. According to the result of the coronary angiography, all participants were divided into two groups: non-CAD group (*n* = 66) and CAD group (*n* = 79). The CAD group was further divided into single-vessel (*n* = 25), double-vessel (*n* = 30) and triple-vessel (*n* = 24) disease groups in line with different lesioned vessels of CAD. Plasma CTRP 3 concentration was determined by enzyme-linked immunosorbent assay (ELISA).

**Results:**

Serum levels of CTRP 3 were significantly higher in CAD patients than in non-CAD patients (CAD: 56.68 ± 3.63 ng/ml, non-CAD: 44.10 ± 3.20 ng/ml, *p* < 0.01). Significant differences of CTRP 3 levels were also found between single-vessel group and triple-vessel group (single-vessel group: 44.80 ± 3.14 ng/ml, triple-vessel group: 75.07 ± 9.41 ng/ml, *p* < 0.005). Multiple logistic regression analysis revealed that CTRP 3 levels, together with HDL cholesterol and glucose, correlated with CAD.

**Conclusions:**

Elevated serum CTRP 3 levels were closely related to the prevalence and severity of CAD, suggesting that it might be regarded as a novel biomarker for CAD.

**Electronic supplementary material:**

The online version of this article (doi:10.1186/s12872-017-0646-7) contains supplementary material, which is available to authorized users.

## Background

Given the dramatic changes in lifestyle worldwide, increasing obesity-related disease including type 2 diabetes, dyslipidemia and hypertension have become a serious problem in society. Obesity causes a great deal of metabolic disorders which can culminate in the development of atherosclerotic diseases including coronary artery disease (CAD) [1–4]. Multiple pro-inflammatory cytokines including tumor necrosis factor-a (TNF-a) and IL-6 are up-regulated in cases of obesity and intensify the prevalence and the progression of CAD [5–7].

The complement C1q tumor necrosis factor related protein (CTRP) superfamily is a newly found cluster of adipokines, which share a common structure composed of collagenous and globular C1q-like domains, and 15 members in humans have been identified presently [8, 9]. C1q/tumor necrosis factor related protein-3 (CTRP 3) belongs to the CTRP family, which is abundantly expressed in adipose tissue and chondrocytes [10]. CTRP 3 has been reported for the first time to have comparable potency to APN on vasorelaxation in C57BL mice in 2011 [11]. Circulating CTRP 3 also improves insulin sensitivity and is strongly associated with glucose and lipid metabolism [12]. A recent in vivo study using the CTRP 3 transgenic mice model illustrated that overexpression of CTRP 3 in the obese state can reduce the systemic inflammation [13]. Accumulating evidence has demonstrated that CTRPs have diverse biological influences on the cardiovascular system [14, 15].

CTRP 3, a potent anti-inflammatory adipokine, has attracted increasing attention in its role underlying the pathogenesis of CAD [16]. Recent studies have demonstrated that CTRP 3 exhibits protective properties on cardiac and vascular remodeling in mice [17]. However, there is little research exploring the relevance of CTRP 3 with CAD. Here we conducted a study on serum CTRP 3 and the prevalence or severity of CAD.

## Methods

### Study subjects

One hundred five CAD patients were enrolled from inpatients that underwent coronary angiography at Ruijin Hospital between 2013 and 2015. The criteria of CAD we used was a 50% or greater organic stenosis of at least one major coronary artery, as confirmed by coronary angiogram. With the help of cardiovascular angiographic system, the lesion can be classified into left main, anterior descending, circumflex and right coronary artery lesion. A stenosis ≥50% was considered as CAD, two stenoses ≥50% was considered as double-vessel disease and more than two stenoses was defined as triple-vessel disease. We excluded patients with acute myocardial infarction, congestive heart failure, valvular heart disease, cerebral infarction, thrombotic diseases, severe hepatic and renal dysfunction, infections, hemodialysis and malignancy. With reference to their coronary angiography results, they were divided into a CAD group (*n* = 79) and a non-CAD group (*n* = 66, with normal coronary artery angiography). The CAD group was further grouped into three subgroups by their different coronary lesioned vessels: single-vessel group (*n* = 25), double-vessel group (*n* = 30) and triple-vessel group(*n* = 24). All participants were given written informed consent. This study was approved by the ethics committee of Ruijin Hospital.

### Laboratory methods

Blood samples were obtained from CAD patients and non-CAD subjects after overnight fasting. Plasma CTRP 3 levels were measured quantitatively by enzyme-linked immunosorbent assay using the commercially available ELISA kit (Senxiong Biotech Industry Company, Shanghai, China) and we followed the manufacturer’s recommendations (the detail of the ELISA Assay Procedure is presented in Additional file 1). Other serum indexes were determined by standard assays. Blood pressure (BP) was measured with an appropriate arm cuff and a mercury column sphygmomanometer after at least 10 min’ rest in sitting position. Body mass index (BMI) was calculated as the ratio of weight (kg) to squared height (m^2^).

### Statistical analysis

Statistical analyses were performed using SPSS 17.0 software. Values were presented as mean ± standard error (SE) for continuous variables. The measurement data was conducted by the normality test. The difference of continuous variables was compared using the t test and the Mann-Whitney U test between groups. The count data was assessed using chi-squared test. The Kruskal-Wallis H test was used for multiple comparisons. Bonferroni’s correction was used to adjust for multiple comparisons. Association between CAD and all other parameters was first examined by simple logistic correlation analysis, and then evaluated by multiple logistic regression analysis using parameters selected from single analysis. Logistic regression analysis was used to identify the association between CAD and other parameters. All statistical tests used a *P* value of 0.05 in a two-tail test as statistically significant.

## Results

### Clinical characteristics

**Fig. 1:**
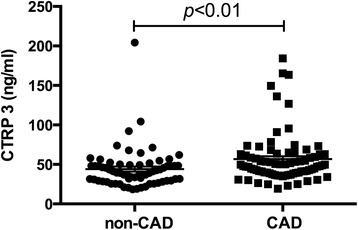
Plasma CTRP 3 levels in non-CAD patients and CAD patients. Plasma concentration of CTRP3 in non-CAD group (*n* = 66) and CAD group (*n* = 79) was measured by ELISA system

Clinical characteristics in CAD and non-CAD groups are displayed in Table 1. Compared with the non-CAD group, patients in the CAD group showed a more frequent proportion of males, smokers and diabetics. We found that CAD patients had significantly higher BMI, systolic BP, fasting glucose and CTRP 3 (Fig. 1) than non-CAD patients. High density lipoprotein-cholesterol (HDL-c) level was lower in the CAD group than that in the non-CAD group. There were no statistical differences in age, frequency of smokers, diastolic BP, triglyceride (TG), total cholesterol (TC), low density lipoprotein-cholesterol (LDL-c) between the two groups.

**Table 1:** Clinical characteristics of participants in CAD and non-CAD groups

Characteristics	Non-CAD (*n* = 66)	CAD (*n* = 79)	*P* value
Male (%)	36 (54.55)	57 (72.15)	<0.05
Age (years)	63.70 ± 1.18	66.95 ± 1.25	0.064
BMI (kg/m^2^)	23.93 ± 0.35	24.94 ± 0.31	<0.05
Smoking (%)	9 (13.64)	11 (13.92)	0.960
Systolic BP (mmHg)	127.42 ± 2.10	134.39 ± 2.21	<0.05
Diastolic BP (mmHg)	75.48 ± 1.23	74.79 ± 1.13	0.681
Glucose (mmol/L)	5.71 ± 0.16	6.97 ± 0.37	<0.01
Diabetes (%)	28 (42.42)	35 (44.30)	0.820
Total cholesterol (mmol/L)	4.40 ± 0.14	4.17 ± 0.12	0.204
LDL cholesterol (mmol/L)	2.69 ± 0.12	2.84 ± 0.09	0.294
HDL cholesterol (mmol/L)	1.19 ± 0.04	1.05 ± 0.03	<0.05
Triglyceride (mmol/L)	1.56 ± 0.13	1.81 ± 0.11	0.129
CTRP 3 (ng/ml)	44.10 ± 3.20	56.68 ± 3.63	<0.01

Data are presented as mean ± SE. *BMI* Body mass index, *BP* Blood pressure, *LDL* Low density lipoprotein, *HDL* High density lipoprotein, *CTRP 3* C1q/TNF-related protein-3

The clinical characteristics of the CAD subgroups are listed in Table 2. There were significant differences in the CTRP 3 levels and proportion of males between these groups. However, there were no statistical differences in age, BMI, diastolic BP, diastolic BP, Glucose, triglyceride (TG), total cholesterol (TC), low density lipoprotein-cholesterol (LDL-c) and high density lipoprotein-cholesterol (HDL-c).

**Table 2:** Clinical characteristics of participants in CAD subgroups

Characteristics	Single-vessel (*n* = 25)	Double-vessel (*n* = 30)	Triple-vessel (*n* = 24)	*P* value
Male (%)	13 (0.52)	23 (0.77)	21 (0.875)	<0.05
Age (years)	68.240 ± 2.27	65.567 ± 1.85	67.33 ± 2.50	0.665
BMI (kg/m^2^)	25.17 ± 0.56	24.74 ± 0.40	24.96 ± 0.67	0.853
Smoking (%)	3 (0.12)	4 (0.13)	4 (0.17)	0.888
Systolic BP (mmHg)	132.72 ± 3.08	133.27 ± 4.22	137.94 ± 3.91	0.643
Diastolic BP (mmHg)	74.96 ± 2.05	74.30 ± 1.92	75.25 ± 1.94	0.939
Glucose (mmol/L)	6.82 ± 0.66	7.34 ± 0.61	6.67 ± 0.68	0.735
Diabetes (%)	11(0.44)	13(0.43)	11 (0.46)	0.983
Total cholesterol (mmol/L)	4.13 ± 0.16	4.18 ± 0.18	4.20 ± 0.26	0.974
LDL cholesterol (mmol/L)	2.76 ± 0.13	2.87 ± 0.15	2.89 ± 0.18	0.843
HDL cholesterol (mmol/L)	1.10 ± 0.07	0.98 ± 0.05	1.09 ± 0.05	0.267
Triglyceride (mmol/L)	1.74 ± 0.14	2.00 ± 0.19	1.67 ± 0.22	0.647
CTRP 3 (ng/ml)	44.80 ± 3.14	51.87 ± 4.02	75.07 ± 9.41	<0.05

Data are presented as mean ± SE. *BMI* Body mass index, *BP* Blood pressure, *LDL* Low density lipoprotein, *HDL* High density lipoprotein, *CTRP 3* C1q/TNF-related protein-3

### Relationship between CTRP 3 and different coronary lesioned vessels of CAD

**Fig. 2:**
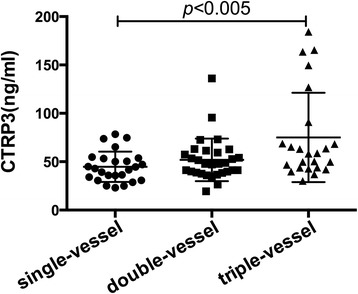
CTRP 3 levels in patients with single-vessel, double-vessel and triple-vessel disease

In order to explore the relationship between serum CTRP 3 and the different coronary lesioned vessels of CAD, CTRP 3 levels in different subgroups were compared. CAD patients were grouped by the different coronary lesioned vessels, the CTRP 3 level in single-vessel, double-vessel and triple-vessel disease group was 44.80 ± 3.14, 51.87 ± 4.02, 75.07 ± 9.41 ng/ml, respectively. Circulating CTRP 3 levels (Fig. 2) were increased with the number of lesioned coronary vessels, and it was significantly different between single-vessel and triple-vessel group (*P* < 0.005). There was no significant difference between both the single-vessel and double-vessel as well as double-vessel and triple-vessel disease groups.

### Association of CTRP 3 levels with CAD

To determine the association between CTRP 3 and CAD, single and multiple logistic regression analyses were performed between CAD and non-CAD groups. In single logistic regression we found that BMI, systolic BP, fasting glucose, HDL-c and CTRP3 were significantly associated with CAD (Table 3). Multiple logistic regression analysis with BMI, systolic BP, fasting glucose, HDL-c and CTRP 3 demonstrated that HDL-c, glucose and CTRP 3 were markedly associated with CAD.

**Table 3:** Association with CAD

	Single		Multiple	
	OR (95%CI)	*P* value	OR (95%CI)	*P* value
Age (years)	1.031 (0.998–1.065)	0.067		
BMI (kg/m^2^)	1.144 (1.011–1.295)	<0.05	1.069 (0.935–1.222)	0.327
Systolic BP (mmHg)	1.021 (1.002–1.040)	<0.05	1.018 (0.998–1.039)	0.071
Diastolic BP (mmHg)	0.993 (0.961–1.026)	0.678		
Glucose (mmol/L)	1.313 (1.066–1.617)	<0.01	1.272 (1.017–1.590)	<0.05
Total cholesterol (mmol/L)	0.817 (0.599–1.116)	0.205		
LDL cholesterol (mmol/L)	1.228 (0.837–1.802)	0.293		
HDL cholesterol (mmol/L)	0.207 (0.063–0.682)	<0.01	0.240 (0.063–0.915)	<0.05
Triglyceride (mmol/L)	1.311 (0.918–1.870)	0.136		
CTRP3 (ng/ml)	1.018 (1.003–1.034)	<0.05	1.015 (1.001–1.031)	<0.05

*SD* Standard deviation, *CI* Confidence intervals, *BMI* Body mass index, *BP* Blood pressure, *LDL* Low density lipoprotein, *HDL* High density lipoprotein, *CTRP 3* C1q/TNF-related protein 3

## Discussion

CTRP 3 is a newly discovered adipokine with anti-inflammatory properties, which may underpin the pathogenesis of obesity-linked diseases including CAD. Several studies have reported the association of circulating CTRP 3 with obesity and CAD in vitro and in vivo [18–20]. Functional knowledge gained from multiple mice models has promoted deep investigations of plasma CTRP 3 levels in humans; however, conflicting results have been shown for its relationship with diabetes and metabolic syndrome [14, 21, 22]. Our study demonstrated that the plasma levels of circulating CTRP 3 are increased in patients with CAD compared to non-CAD subjects, and the CTRP 3 levels positively correlate with the progress of disease. Multiple logistic regression analysis showed that HDL-c, glucose and CTRP 3 level were significantly related with CAD. In contrast, our result disagreed with Reza Fadaei’s study which reported that patients with CAD had markedly decreased circulating CTRP 3 concentration [23]. A defensive response to counteract the metabolic stress or resistance to CTRP3 action might account for our paradoxical increase of serum CTRP 3.

CTRP 3 exerts protective effects on the cardiovascular system by promoting angiogenesis and preventing unbalanced cardiovascular remodeling. Yi first demonstrated that the replenishment of CTRP 3 effectively promotes post-ischemic angiogenesis by elevating cardiomyocyte-endothelial cell communication and attenuates adverse remodeling in C57/BL6 mice with coronary artery occlusion [24]. Recently, Li found the CTRP 3/cartducin gene could be transiently up-regulated in a balloon-injured rat carotid artery tissue during a period characterized by neointimal formation [25]. All these observations proved that CTRP 3 levels were closely related to the pathogenesis of CAD. Consistent with these findings, our data in this study indicated that CTRP 3 levels in CAD group were much higher than non-CAD group, and may contribute to the defensive responses protecting the cardiovascular system. Circulating CTRP 9 had been demonstrated to be associated with the severity of CAD. However, there is little research about the relationship of CTRP 3 levels with CAD [15].

There were several limitations to this study. First, the sample size was small and most of the study subjects were from Shanghai Province where people have similar lifestyles. These results might not be generalizable to populations in other areas. Secondly, cholesterol-lowering statin medications which were reported to play important roles in the development of atherosclerosis were not taken into account in this study [26, 27]. Third, this was a cross-sectional study, which limited our ability to conclude a cause-and-effect relationship between the CTRP3 and CAD. Future studies conducted on a larger population that exclude potential confounding factors such as medication treatment history are needed.

## Conclusion

In conclusion, we have demonstrated that serum CTRP 3 levels are elevated in CAD patients than non-CAD patients, and the CTRP 3 levels positively correlate with the progress of the disease. These results indicate that CTRP 3 may play an important role in the pathophysiology of CAD, suggesting that CTRP 3 may represent a novel biomarker for CAD.

## Additional file

Additional file 1: Method S1. ELISA Assay Procedure. (DOCX 135 kb)

## Abbreviations

BMI: Body mass index
CAD: Coronary artery disease
CTPR 3: Complement C1q tumor necrosis factor related protein-3
HDL: High density lipoprotein-cholesterol
LDL: Low density lipoprotein-cholesterol
TC: Total cholesterol
TG: Triglyceride
