# Supplementary material for: Association of serum C1q/TNF-related protein-3 (CTRP-3) in patients with coronary artery disease
Source: BMC Cardiovasc Disord. 2017 Jul 28;17:210. doi: 10.1186/s12872-017-0646-7 (PMC5534082; doi:10.1186/s12872-017-0646-7)
Supplement: Additional file 1: — Method S1. ELISA Assay Procedure. (DOCX 135 kb) [file 12872_2017_646_MOESM1_ESM.docx]

ELISA Assay Procedure:

1. Bring all reagents and samples to room temperature before use. all standards, samples and controls were assayed in duplicate.
2. Unused well strips should be returned to the plate packet and stored at 4 ℃.
3. Standard Dilution Preparation: Add 50ul of Assay Dilution to each well and prepare serially diluted standards immediately prior to use according to the manufacture’s instruction.
4. Add 50ul of each standard and sample into appropriate wells. Cover well and incubate for 30min at 37℃ with gentle shaking.
5. Discard the solution and wash 4 times with 1X Wash Solution. Wash by filling each well with 1X Wash Solution (300μL) using a multi-channel Pipette or autowasher. Complete removal of liquid at each step is essential to good performance. After the last wash, remove any remaining 1X Wash Buffer by aspirating or decanting. Invert the plate and blot it against clean paper towels.
6. Add 50ul of CTRP 3 Conjugate to each well. Cover well and incubate for 30min at 37℃.
7. Discard the solution. Repeat the wash as in the step 5.
8. Add 50ul of chromogenic agent A and 50ul of chromogenic agent B separately to each well with gentle shaking. Cover well and incubate for 15min at 37℃.
9. Add 50ul of Stop Solution to each well (The color in the wells should change from blue to yellow). Determine the optical density of each well immediately using a microplate reader set to 450nm.
